# Association of microRNA polymorphisms with the risk of head and neck squamous cell carcinoma in a Chinese population: a case–control study

**DOI:** 10.1186/s40880-016-0136-9

**Published:** 2016-08-11

**Authors:** Limin Miao, Lihua Wang, Longbiao Zhu, Jiangbo Du, Xun Zhu, Yuming Niu, Ruixia Wang, Zhibin Hu, Ning Chen, Hongbing Shen, Hongxia Ma

**Affiliations:** 1 Jiangsu Key Laboratory of Oral Diseases, Nanjing Medical University, Nanjing, 210029 Jiangsu P. R. China; 2 Department of Epidemiology and Biostatistics, School of Public Health, Nanjing Medical University, Nanjing, 211166 Jiangsu P. R. China; 3 Department of Stomatology and Center for Evidence-Based Medicine and Clinical Research, Taihe Hospital, Hubei University of Medicine, Shiyan, 442000 Hubei P. R. China; 4 Jiangsu Key Lab of Cancer Biomarkers, Prevention and Treatment, Collaborative Innovation Center for Cancer Personalized Medicine, Nanjing Medical University, Nanjing, 211166 Jiangsu P. R. China

**Keywords:** Head and neck cancer, microRNA, Polymorphism, Squamous cell carcinoma, Susceptibility

## Abstract

**Background:**

MicroRNA (miRNA) polymorphisms may alter miRNA-related processes, and they likely contribute to cancer susceptibility. Various studies have investigated the associations between genetic variants in several key miRNAs and the risk of human cancers; however, few studies have focused on head and neck squamous cell carcinoma (HNSCC) risk. This study aimed to evaluate the associations between several key miRNA polymorphisms and HNSCC risk in a Chinese population.

**Methods:**

In this study, we genotyped five common single-nucleotide polymorphisms (SNPs) in several key miRNAs (*miR*-*149* rs2292832, *miR*-*146a* rs2910164, *miR*-*605* rs2043556, *miR*-*608* rs4919510, and *miR*-*196a2* rs11614913) and evaluated the associations between these SNPs and HNSCC risk according to cancer site with a case–control study including 576 cases and 1552 controls, which were matched by age and sex in a Chinese population.

**Results:**

The results revealed that *miR*-*605* rs2043556 [dominant model: adjusted odds ratio (OR) 0.71, 95% confidence interval (CI) 0.58–0.88; additive model: adjusted OR 0.74, 95% CI 0.62–0.89] and *miR*-*196a2* rs11614913 (dominant model: adjusted OR 1.36, 95% CI 1.08–1.72; additive model: adjusted OR 1.28, 95% CI 1.10–1.48) were significantly associated with the risk of oral squamous cell carcinoma (OSCC). Furthermore, when these two loci were evaluated together based on the number of putative risk alleles (rs2043556 A and rs11614913 G), a significant locus-dosage effect was noted on the risk of OSCC (*P*_trend_ < 0.001). However, no significant association was detected between the other three SNPs (*miR*-*149* rs2292832, *miR*-*146a* rs2910164, and *miR*-*608* rs4919510) and HNSCC risk.

**Conclusion:**

Our study provided the evidence that *miR*-*605* rs2043556 and *miR*-*196a2* rs11614913 may have an impact on genetic susceptibility to OSCC in Chinese population.

## Background

Head and neck cancer, predominantly head and neck squamous cell carcinoma (HNSCC), represents a common cancer worldwide and has been considered a serious and growing public health problem in many countries [1, 2]. It was estimated that 45,780 new patients would be diagnosed with cancer of the oral cavity and the pharynx, and 8650 deaths from these diseases occurred in 2015 in the United States alone [3]. Environmental carcinogens and carcinogenic viruses have been identified as the main etiologic factors for HNSCC [4]. Furthermore, genetic variants play a risk-modulating role in the etiology of HNSCC [5].

MicroRNAs (miRNAs) are 20–24 nt single-stranded RNA molecules that repress the expression of specific target genes by binding to the 3′-untranslated regions (UTRs) of messenger RNA (mRNA) [6]. A single miRNA may regulate the expression of many genes, and it has been proposed that more than one-third of all protein-coding genes are under translational control by miRNAs [7]. Numerous studies have demonstrated that aberrant expression of miRNAs is closely associated with the cell proliferation, invasion, metastasis, and prognosis of various cancers [8, 9]. Given that small variations in the expression of a specific miRNA may affect thousands of target mRNAs and result in diverse functional consequences [10], miRNAs have been considered ideal candidate genes for cancer predisposition.

Studies have demonstrated that potentially functional single nucleotide polymorphisms (SNPs) located in several key miRNAs may influence the function of mature miRNAs and then affect the process of carcinogenesis [11–13]. For example, rs2292832 in *miR*-*149* and rs2043556 in *miR*-*605* were associated with the modified expression level of these two miRNAs [14]. rs2910164 in *miR*-*146a* altered the mature *miR*-*146a* expression level that was involved in the regulation of cell differentiation and cancer formation [15, 16]. rs4919510 in *miR*-*608* has been predicted by in silico algorithms to exhibit differential capacities to bind to the potential target genes of *miR*-*608*, such as the insulin receptor (*IR*) and tumor protein 53 (*TP53*) [17]. Furthermore, rs11614913 in *miR*-*196a2* affects the expression of *miR*-*196a*, and aberrant regulation of *miR*-*196a* is involved in the development and progression of several cancers, including oral cancer [18]. To date, some population studies and meta-analyses have been performed to investigate the associations between polymorphisms of the above important miRNAs and the risk of multiple types of malignant tumors [19, 20]. However, the results were inconsistent, and few studies focused on the associations of these SNPs with HNSCC risk in Chinese population.

Thus, we performed a case-control study on associations of five common SNPs in key miRNAs (rs2292832 in *miR*-*149*, rs2910164 in *miR*-*146a*, rs2043556 in *miR*-*605*, rs4919510 in *miR*-*608*, and rs11614913 in *miR*-*196a2*) with HNSCC risk in China.

## Methods

### Study subjects

This study is a hospital-based case–control study. All newly diagnosed HNSCC patients historically confirmed by two pathologists were consecutively recruited from Jiangsu Stomatological Hospital and the First Affiliated Hospital of Nanjing Medical University, Nanjing, China between January 2009 and May 2013. Exclusion criteria included secondary HNSCC or metastasized cancer from other organs. None of the patients received neoadjuvant chemotherapy or radiotherapy before surgery. Cancer-free controls matched to the cases by age (±5 years) and sex were randomly selected from a cohort of more than 30,000 participants in a community-based screening program for non-infectious diseases in the Jiangsu Province, China. All participants were genetically unrelated and of the ethnic Han Chinese population. Each participant was scheduled for a face-to-face interview to answer a structured questionnaire that elicited information on demographic characteristics and environmental exposure history, such as age, sex, smoking status, and drinking status. Written informed consent was obtained from each participant, and the study was approved by the Institutional Review Boards of all relevant institutes.

### SNP selection and genotyping

Based on previous reports about miRNA polymorphisms and cancer risk [14–18], we chose five most investigated and potentially functional SNPs (rs2292832 in *miR*-*149*, rs2910164 in *miR*-*146a*, rs2043556 in *miR*-*605*, rs4919510 in *miR*-*608*, and rs11614913 in *miR*-*196a2*) for genotyping. Venous blood was collected from all subjects and centrifuged at a speed of 4000 round/min for 10 min. The centrifuged blood was stored at −40 °C for use. Genomic DNA was isolated from leukocyte pellets of venous blood by proteinase K digestion, and this process was followed by phenol chloroform extraction. All DNA samples were assessed for quality and quantity using Nanodrop (Thermo Scientific, Waltham, MA, USA) and DNA electrophoresis (agarose gel imaging system, agarose gel electronic balance, and electronic tank supplied by Oxoid company, Basingstoke, England; micropipette, microwave oven, and electrophoresis apparatus supplied by Gilson company, Madison, WI, USA) before genotyping. SNPs were genotyped by using Illumina Infinium1 Human Exome BeadChip (Illumina Inc., San Diego, CA, USA), and genotype calling was performed using the GenTrain version 1.0 clustering algorithm in GenomeStudio V2011.1 (Illumina). The overall call rate was 99.77%–99.91% for all SNPs.

### Statistical analysis

The Hardy–Weinberg equilibrium was tested by a goodness-of-fit χ^2^ test to compare the observed genotype frequencies with the expected ones among the control subjects. Distributions of selected demographic variables, risk factors, and frequencies of variant genotypes between the cases and controls were evaluated by using the Pearson’s Chi squared test (uncorrected). The associations of variant genotypes with HNSCC risk were estimated by computing odds ratios (ORs) and 95% confidence intervals (CIs) from both univariate and multivariate logistic regression analyses according to cancer site. The heterogeneity between subgroups was assessed with the Chi square-based *Q* test. All statistical analyses were performed with Statistical Analysis System software (v.9.1 SAS Institute, Cary, NC, USA). *P* < 0.05 was considered as the level of statistical significance.

Additionally, we used another data-mining tool, the non-parametric multifactor dimensionality reduction (MDR) software (version 2.0 beta 8.4, Norris-Cotton Cancer Center, Geisel School of Medicine, Dartmouth College, Hanover, NH, USA) to identify the potential locus–locus and gene-environment interactions with trichotomies genotypes, age (dichotomized into ≥60 years and <60 years), sex, smoking status, and drinking status. The fitness of the MDR model was assessed by estimating the testing accuracy and the cross-validation consistency (CVC). Models that were true positive would have estimating the testing accuracy of >0.5. The best model with the highest CVC and the highest testing accuracy was selected [21].

## Results

### Selected characteristics of studied subjects

A total of 576 HNSCC patients and 1552 cancer-free controls were included in the study. Distributions of physiological characteristics in the case and control groups are presented in Table 1. No significant difference in the distributions of age, sex, and smoking status were noted between the case and control groups. Expectedly, more drinkers were found in the case group than in the control group (44.3 vs. 32.8%, *P* < 0.001). Further, logistic regression suggested that drinking status was associated with an increased HNSCC risk (β = 0.493, OR 1.64, 95% CI 1.35–1.99, *P* < 0.001). Although the proportion of smokers was a bit higher in the case group (45.3%) than in the control group (42.6%), the association between smoking and HNSCC risk was not significant (β = 0.111, OR 1.12, 95% CI 0.92–1.35, *P* = 0.260). In the 576 cases, 462 (80.2%) had oral squamous cell carcinoma (OSCC), and 114 (19.8%) had HNSCC at other sites [9 (1.6%) had oropharyngeal tumor, 102 (17.7%) had laryngeal tumor, 1 had nasal sinus cancer, 1 had parotid carcinoma, and 1 had salivary gland carcinoma].

**Table 1:** Selected characteristics of head and neck squamous cell carcinoma (HNSCC) patients and cancer-free controls

Variable	Patients [cases (%)]	Controls [cases (%)]	*P* ^a^
Total	576	1552	
Age (years)			
<60	265 (46.0)	719 (46.3)	0.895
≥60	311 (54.0)	833 (53.7)	
Gender			0.750
Female	214 (37.2)	565 (36.4)	
Male	362 (62.8)	987 (63.6)	
Smoking status			0.260
No	315 (54.7)	891 (57.4)	
Yes	261 (45.3)	661 (42.6)	
Drinking status			*<0.001*
No	321 (55.7)	1043 (67.2)	
Yes	255 (44.3)	509 (32.8)	

Italic value indicate significance of *p* value (*p* < 0.05)

^a^Two-sided Chi squared test

### Primary information of selected SNPs

The position, minor allele frequencies (MAFs), and other primary information of five selected SNPs are presented in Table 2. The Hardy–Weinberg equilibrium was not severely violated judging from the goodness-of-fit χ^2^ test (all *P* > 0.05). Among the five loci, the genotype distributions of two SNPs were significantly different between the case and control groups (*P* = 0.004 for *miR*-*605* rs2043556 and *P* = 0.019 for *miR*-*196a2* rs11614913).

**Table 2:** Primary information and minor allele frequencies (MAFs) of selected single-nuclide polymorphisms (SNPs)

Gene	Chromosome	SNP	Base change	Call rates (%)	HWE	MAF in controls	*P* ^a^	*P* ^b^
Has-miR-149	2q37.3	rs2292832	A>G	99.77	0.092	0.322	0.349	0.436
Pre-miR-146a	5q34	rs2910164	G>C	99.81	0.468	0.429	0.558	0.558
Has-miR-605	10q21.1	rs2043556	A>G	99.77	0.753	0.281	*0.004*	*0.020*
Has-miR-608	10q25.1	rs4919510	G>C	99.85	0.835	0.425	0.245	0.408
Pre-miR-196a	12q13.13	rs11614913	A>G	99.91	0.796	0.432	*0.019*	*0.048*

Italic value indicate significance of *p* value (*p* < 0.05)

*HWE* Hardy–Weinberg equilibrium, *MAF* minor allele frequency

^a^Two-sided Chi squared test for the comparison of the allele frequency between HNSCC patients and cancer-free controls

^b^ *P* values adjusted by false discovery rate (FDR) method

### Associations between selected SNPs and HNSCC risk

Logistic regression analyses revealed that variant genotypes of *miR*-*605* rs2043556 significantly decreased the risk of OSCC (AG vs. AA: adjusted OR 0.74, 95% CI 0.59–0.93; GG vs. AA: adjusted OR 0.56, 95% CI 0.35–0.89; dominant model: adjusted OR 0.71, 95% CI 0.58–0.88; recessive model: adjusted OR 0.63, 95% CI 0.40–1.00; additive model: adjusted OR 0.74, 95% CI 0.62–0.89), whereas variant genotypes of rs11614913 in *miR*-*196a2* significantly increased the risk of OSCC (GG vs. AA: adjusted OR 1.64, 95% CI 1.22–2.21; dominant model: adjusted OR 1.36, 95% CI 1.08–1.72; recessive model: adjusted OR 1.42, 95% CI 1.11–1.83; additive model: adjusted OR 1.28, 95% CI 1.10–1.48) (Table 3). After false discovery rate (FDR) adjustment, the above associations remained significant for rs2043556 in *miR*-*605* (AG vs. AA: *P* = 0.045; GG vs AA: *P* = 0.038; dominant model: *P* = 0.010; additive model: *P* = 0.005) and rs11614913 in *miR*-*196a2* (GG vs. AA: *P* = 0.005; dominant model: *P* = 0.025; recessive model: *P* = 0.030; additive model: *P* = 0.003). We also performed logistic regression analysis conditioning on all selected miRNAs and SNPs, and the results indicated that the effects of rs2043556 in *miR*-*605* and rs11614913 in *miR*-*196a2* on OSCC risk were independent (*P* = 0.001 for both *miR*-*605* rs2043556 and *miR*-*196a2* rs11614913 in additive model).

**Table 3:** Logistic regression analysis for associations between selected SNPs and HNSCC risk

SNP	Genotype^a^	Controls [number (%)]	Oral cancer patients [number (%)]	Adjusted OR (95% CI)^b^	*P* ^b^	*P* ^c^	Non-oral cancer patients [number (%)]	Adjusted OR (95% CI)^b^	*P* ^b^
***miR***-***605 rs2043556***	AA	798 (51.6)	278 (60.3)	1.00			55 (48.2)	1.00	
	AG	631 (40.8)	160 (34.7)	*0.74 (0.59*–*0.93)*	*0.009*	*0.045*	52 (45.6)	1.19 (0.80–1.78)	0.396
	GG	119 (7.7)	23 (5.0)	*0.56 (0.35*–*0.89)*	*0.015*	*0.038*	7 (6.1)	0.85 (0.38–1.94)	0.708
	Dominant model	NA	NA	*0.71 (0.58*–*0.88)*	*0.002*	*0.010*	NA	1.14 (0.77–1.67)	0.518
	Recessive model	NA	NA	*0.63 (0.40*–*1.00)*	*0.050*	0.125	NA	0.79 (0.36–1.75)	0.561
	Additive model	NA	NA	*0.74 (0.62*–*0.89)*	*0.001*	*0.005*	NA	1.04 (0.77–1.42)	0.787
***miR***-***196a2 rs11614913***	AA	503 (32.5)	122 (26.4)	1.00			40 (35.1)	1.00	
	AG	755 (48.7)	228 (49.4)	1.25 (0.98–1.61)	0.075	0.188	56 (49.1)	0.93 (0.61–1.43)	0.736
	GG	292 (18.8)	112 (24.2)	*1.64 (1.22*–*2.21)*	*0.001*	*0.005*	18 (15.8)	0.76 (0.43–1.37)	0.366
	Dominant model	NA	NA	*1.36 (1.08*–*1.72)*	*0.010*	*0.025*	NA	0.88 (0.59–1.33)	0.547
	Recessive model	NA	NA	*1.42 (1.11*–*1.83)*	*0.006*	*0.030*	NA	0.80 (0.47–1.35)	0.402
	Additive model	NA	NA	*1.28 (1.10*–*1.48)*	*0.001*	*0.003*	NA	0.88 (0.67–1.17)	0.386
*miR*-*149* rs2292832	AA	726	226	1.00			57	1.00	
	AG	647	193	0.96 (0.77–1.19)	0.696	0.696	38	0.76 (0.49–1.17)	0.206
	GG	175	42	0.76 (0.52–1.10)	0.141	0.235	19	1.37 (0.79–2.39)	0.268
	Dominant model	NA	NA	0.91 (0.74–1.13)	0.399	0.499	NA	0.89 (0.60–1.31)	0.556
	Recessive model	NA	NA	0.77 (0.54–1.10)	0.156	0.260	NA	1.55 (0.91–2.62)	0.107
	Additive model	NA	NA	0.90 (0.77–1.06)	0.198	0.248	NA	1.05 (0.79–1.39)	0.735
*miR*-*146a* rs2910164	GG	497	154	1.00			40		
	GC	773	228	0.95 (0.75–1.21)	0.685	0.861	53	0.82 (0.53–1.27)	0.376
	CC	278	80	0.93 (0.68–1.27)	0.656	0.656	21	0.90 (0.51–1.57)	0.702
	Dominant model	NA	NA	0.95 (0.76–1.18)	0.633	0.633	NA	0.84 (0.56–1.27)	0.407
	Recessive model	NA	NA	0.96 (0.73–1.27)	0.771	0.771	NA	1.01 (0.61–1.66)	0.975
	Additive model	NA	NA	0.96 (0.83–1.12)	0.629	0.629	NA	0.93 (0.70–1.23)	0.589
*miR*-*608* rs4919510	AA	509	137	1.00			40		
	AG	762	232	1.14 (0.90–1.45)	0.283	0.472	53	0.85 (0.55–1.31)	0.464
	GG	278	93	1.23 (0.91–1.67)	0.179	0.224	21	0.97 (0.56–1.70)	0.927
	Dominant model	NA	NA	1.17 (0.93–1.46)	0.187	0.312	NA	0.88 (0.59–1.32)	0.546
	Recessive model	NA	NA	1.14 (0.87–1.48)	0.345	0.431	NA	1.07 (0.65–1.77)	0.787
	Additive model	NA	NA	1.11 (0.96–1.29)	0.160	0.267	NA	0.96 (0.73–1.28)	0.794

Italic value indicate significance of *p* value (*p* < 0.05)

*NA* not available

^a^ *miR*-*605* rs2043556 was genotyped in 575 cases and 1548 controls; *miR*-*196a2* was genotyped in 576 cases and 1550 controls; *miR*-*149* rs2292832 was genotyped in 575 cases and 1548 controls; *miR*-*146a* rs2910164 was genotyped in 576 cases and 1548 controls; and *miR*-*608* rs4919510 was genotyped in 576 cases and 1549 controls

^b^Adjusted by age, sex, smoking status, and drinking status

^c^ *P* values of multiple comparisons for false discovery rate using the FDR method (*n* = 5, refer to the number of SNPs)

### Combined effects of the two significant SNPs on OSCC risk

When these two loci were evaluated together by the number (0–4) of putative risk alleles (*miR*-*605* rs2043556 A, A and *miR*-*196a2* rs11614913 G, G), a significant locus-dosage effect was detected on HNSCC risk between the groups with 0–2 risk alleles and 3–4 risk alleles (*P*_trend_ < 0.001). Compared with the group with 0–1 risk allele, the groups with 3 and 4 risk alleles had significantly increased risk of OSCC with adjusted ORs of 1.51 (95% CI 1.10–2.09) and 2.23 (95% CI 1.51–3.29) (Table 4). Compared with the risk in the groups with 0–2 risk alleles, the increase in OSCC risk remained significant for the group with 3–4 risk alleles (adjusted OR 1.48, 95% CI 1.20–1.83). Logistic regression analyses identified no association between the other three SNPs and OSCC risk (data not shown).

**Table 4:** Combined effects of *miR*-*605* rs2043556 and *miR*-*196a2* rs11614913 on oral squamous cell carcinoma (OSCC) risk

Number of risk alleles^a^	Patients [number (%)]	Controls [number (%)]	Adjust OR (95% CI)^b^	*P* ^b^
0–1	66 (14.3)	303 (19.6)	1.00	
2	153 (33.2)	575 (37.2)	1.20 (0.87–1.66)	0.262
3	168 (36.4)	517 (33.4)	*1.51 (1.10*–*2.09)*	*0.011*
4	74 (16.1)	151 (9.8)	*2.23 (1.51*–*3.29)*	<*0.001*
Trend	NA	NA	*1.21 (1.10*–*1.32)*	<*0.001*
Binary classification				<*0.001*
0–2	219 (47.5)	878 (56.8)	1.00	
3–4	242 (52.5)	668 (43.2)	*1.48 (1.20*–*1.83)*	

Italic value indicate significance of *p* value (*p* < 0.05)

^a^The *miR*-*605* rs2043556 A and *miR*-*196a2* rs11614913 G allele were assumed as risk alleles based on the main effect of the individual locus and were genotyped in the 461 OSCC cases and 1546 controls

^b^Adjusted by age, sex, smoking status, and drinking status

### Stratification analysis for association between variant genotypes and OSCC risk

We further conducted a stratification analysis by age, sex, smoking status, drinking status, and tumor site on the associations between rs2043556 in *miR*-*605* and rs11614913 in *miR*-*196a2* and OSCC risk. As presented in Table 5, the association of decreased OSCC risk with *miR*-*605* rs2043556 was more notable in males, whereas the association of increased risk with *miR*-*196a2* rs11614913 was more pronounced in females, non-smokers, and non-drinkers than in their counterparts. The combined effect of rs2043556 in *miR*-*605* and rs11614913 in *miR*-*196a2* on OSCC risk was stronger in patients of ≥60 years old than in those of <60 years old.

**Table 5:** Stratification analysis for association between variant genotypes and OSCC risk

Variable	*miR*-*605* rs2043556 genotype (GG/AG/AA)^a^		Adjusted OR (95% CI)^b^	*P* ^b^	*miR*-*196a2* rs11614913 genotype (GG/AG/AA)^a^		Adjusted OR (95% CI)^b^	*P* ^b^	Combined effect (0-2/3-4 risk alleles)^c^		Adjusted OR (95% CI)^b^	*P* ^b^
	Cancer patients (number)	Controls (number)			Cancer patients (number)	Controls (number)			Cancer patients (number)	Controls (number)		
Age (years)												
<60	10/75/125	55/296/366	*0.76 (0.59*–*1.00)*	*0.042*	56/98/57	135/352/230	*1.33 (1.07*–*1.66)*	*0.011*	102/105	398/317	1.32 (0.97–1.81)	0.081
≥60	13/85/153	64/335/432	*0.73 (0.58*–*0.93)*	*0.011*	56/130/65	157/403/273	*1.24 (1.01*–*1.52)*	*0.038*	117/137	480/351	*1.62 (1.22*–*2.16)*	*0.001*
Sex												
Female	12/68/124	41/227/296	0.78 (0.60–1.02)	0.068	59/99/46	93/275/197	*1.64 (1.30*–*2.07)*	<*0.001*	97/107	331/233	*1.54 (1.11*–*2.12)*	*0.010*
Male	11/92/154	78/404/502	*0.72 (0.57*–*0.91)*	*0.005*	53/129/76	199/480/306	1.08 (0.89–1.32)	0.434	122/135	547/435	*1.47 (1.11*–*1.94)*	*0.008*
Smoking												
Never	15/99/160	70/363/456	*0.79 (0.63*–*0.99)*	*0.037*	74/129/72	172/430/288	*1.32 (1.09*–*1.60)*	*0.004*	135/139	503/385	*1.36 (1.03*–*1.79)*	*0.028*
Ever	8/61/118	49/268/342	*0.66 (0.50*–*0.89)*	*0.006*	38/99/50	120/325/215	1.25 (0.97–1.59)	0.081	84/103	375/283	*1.72 (1.22*–*2.42)*	*0.002*
Drinking												
Never	14/97/161	78/427/534	*0.76 (0.60*–*0.95)*	*0.016*	72/134/67	202/505/335	*1.38 (1.14*–*1.67)*	*0.001*	130/142	582/456	*1.46 (1.11*–*1.92)*	*0.006*
Ever	9/63/117	41/204/264	*0.70 (0.53*–*0.93)*	*0.014*	40/94/55	90/250/168	1.18 (0.93–1.51)	0.175	89/100	296/212	*1.58 (1.12*–*2.22)*	*0.009*

Italic value indicate significance of *p* value (*p* < 0.05)

^a^These data are presented as the numbers of cases with genotypes GG, AG, or AA

^b^Adjusted by age, sex, smoking status, and drinking status

^c^These data are presented as the numbers of cases with 0–2 or 3–4 risk alleles

### MDR analysis for OSCC risk predication

In addition, the MDR method was used to assess potential locus–locus and gene-environment interactions with five SNPs and age, sex, smoking status, and drinking status. As shown in Table 6, age was the strongest factor for predicting HNSCC risk with the highest CVC (100%) and testing accuracy (55.70%). We also observed that the four-factor model, which included age, *miR*-*146a* rs2910164, *miR*-*608* rs4919510, and *miR*-*196a2* rs11614913, was the most accurate model with a testing accuracy of 54.91% and a perfect CVC of 10. However, the two-factor and three-factor models had decreased CVCs, suggesting the models were not very accurate.

**Table 6:** Multifactor dimensionality reduction (MDR) analysis for OSCC risk predication

Best model	Training bal. acc.	Testing bal. acc.	*P* ^a^	CVC
One-factor (age)	0.6063	0.5570	0.1602	10/10
Two-factor (age and *miR*-*605* rs2043556)	0.6575	0.5590	0.1511	5/10
Three-factor (age, *miR*-*146a* rs2910164, and *miR*-*196a2* rs11614913)	0.7276	0.5314	0.4463	6/10
Four-factor (age, *miR*-*146a* rs2910164, *miR*-*608* rs4919510, and *miR*-*196a2* rs11614913)	0.8221	0.5491	0.2411	10/10

*Training bal. acc.* training balanced accuracy, *Testing bal. acc.* testing balanced accuracy, *CVC* cross-validation consistency

^a^ *P* values for testing balanced accuracy

## Discussion

In this case–control study, we examined associations between five common SNPs in miRNAs (*miR*-*149* rs2292832, *miR*-*146a* rs2910164, *miR*-*605* rs2043556, *miR*-*608* rs4919510, and *miR*-*196a2* rs11614913) and HNSCC risk. The results revealed that rs2043556 in *miR*-*605* and rs11614913 in *miR*-*196a2* were significantly associated with OSCC risk in a Chinese population. However, no notable association was detected between other selected SNPs and HNSCC risk.

Once activated, the tumor suppressor *p*53 selectively modulates the expression of target genes involved in cell cycle arrest, apoptosis, and DNA repair [22]. A recent study indicated that *miR*-*605* was a new component in the *p53* gene network [23]. This network is transcriptionally activated by *p53* and post-transcriptionally repressed by murine double minute 2 (*Mdm2*), which inhibits the function of *p53*. Thus, a positive feedback loop is created that aids in the rapid accumulation of *p53* to facilitate its function in response to stress [23]. Id Said et al. [24] reported that high expression of *miR*-*605* could result in a significant reduction in cell viability, clonogenicity, and cell migration in *TP53*-mutant cell types and that rs2043556-variant G allele could significantly result in a decreased expression of *miR*-*605*. Several studies have investigated the associations between *miR*-*605* rs2043556 and cancer risk, and a recent meta-analysis concluded that *miR*-*605* rs2043556 was associated with a significant overall risk of human cancer [25]. In this study, we first examined the effect of *miR*-*605* rs2043556 on the risk of HNSCC and identified a significant linkage between this SNP and the decreased risk of OSCC in a Chinese population. Thus, we hypothesize that *miR*-*605* rs2043556 may affect the expression of *miR*-*605* and the risk of OSCC, which may provide a visual cue regarding the role of this SNP in the development of OSCC.

Rs11614913, which is located at *miR*-*196a2,* impacts the expression of *miR*-*196a2* and is involved in the carcinogenesis of different types of cancer [17, 26, 27]. For example, Tian et al. [28] reported that *miR*-*196a2* rs11614913 was associated with the increased risk of non-small cell lung cancer and poor patient survival, and Hu et al. [29] reported its association with the increased risk of breast cancer. It was also reported that *miR*-*196a2* rs11614913 influenced mature *miR*-*196a* expression (but not the *pre*-*miR*-*196a2* level) and affected the binding ability of *miR*-*196a*-*3p* to its targets [27]. Additionally, Hoffman et al. [30] demonstrated that mature *miR*-*196a2* level was increased 9.3-fold in breast cancer cells transfected with *pre*-*miR*-*196a2*-C (rs11614913), but the levels were only increased 4.4-fold in cells transfected with *pre*-*miR*-*196a2*-T. Such associations were then further supported by studies on other types of cancers. A recent meta-analysis revealed that *miR*-*196a2* rs11614913 was associated with cancer risk, especially risks of lung, colorectal, and breast cancers among Asian populations [31]. Specially, a few studies have investigated the association of rs11614913 in *miR*-*196a2* with HNSCC risk in Caucasian populations, but the results were inconclusive. Liu et al. [32] found no association between *miR*-*196a2* rs11614913 and risk of HNSCC, whereas Christensen et al. [33] reported that the *miR*-*196a2* rs11614913 CC genotype was related with an increased HNSCC risk. Another study identified a significant association between rs11614913 and *miR*-*196a2* expression levels in tumor tissues from OSCC patients, but no association of *miR*-*196a2* rs11614913 with OSCC risk was noted [17]. In this study, we demonstrated that the *miR*-*196a2* rs11614913 G allele was significantly associated with an increased OSCC risk, which is consistent with the study by Christensen et al. [33]. The difference between our study and the other two studies [32, 33] may due to different ethnic backgrounds and different composition of cases. The MAF in our controls was 0.432, whereas it was either 0.420 [32] or not obtained [33] in the literature. Furthermore, the proportion of oral cancer was much higher in our study (80.2%) than that in the other two studies (29.4% and 55.6%, respectively). Larger studies with different ethnic backgrounds and functional investigation are needed to validate these findings.

Studies on associations between the other three SNPs (rs2292832 in *miR*-*149*, rs2910164 in *miR*-*146a*, and rs4919510 in *miR*-*608*) and cancer risk were inconsistent [34–38]. A recent meta-analysis of 12 studies, including 5937 cases and 6081 controls, revealed that *miR*-*149* rs2292832 was not associated with cancer risk [39]. Additionally, only two studies investigated the effect of *miR*-*149* rs2292832 on HNSCC risk, and neither produced significant results [32, 40]. A meta-analysis of 66 case–control studies reported that *miR*-*146a* rs2910164 was a risk factor for HNSCC, which included four studies from a Caucasian population and one study from a Chinese population [41]. However, the results from the Chinese population indicated that *miR*-*146a* rs2910164 was not significantly associated with oral cancer risk [40]. To date, two studies have focused on the associations of *miR*-*608* rs4919510 and cancer risk: one on colorectal cancer [38] and another on breast cancer [37], and their results were inconsistent. In our study, the results demonstrated that none of these three SNPs (rs2292832 in *miR*-*149*, rs2910164 in *miR*-*146a*, and rs4919510 in *miR*-*608*) contributed to the risk of HNSCC in a Chinese population. Given heterogeneous genetic backgrounds in different populations, these findings must be validated in further larger studies.

Several potential limitations of the present study warrant consideration. First, a relatively small sample size may limit the statistical power of our study, especially in the stratification analysis. We made multiple testing adjustments using the FDR method, and the results indicate that the associations between SNPs and OSCC risk remained significant. However, the effect of *miR*-*605* rs2043556 on HNSCC risk was borderline significant after the FDR correction. Thus, our results must be confirmed in further studies. Second, our study is a hospital-based, case–control study, and inherent selection bias cannot be completely excluded. Third, the functional significance of rs2043556 in *miR*-*605* and rs11614913 in *miR*-*196a2* for the development of HNSCC remains largely unknown.

In summary, we identified that *miR*-*605* rs2043556 and *miR*-*196a2* rs11614913 were associated with OSCC risk in a Chinese population. Further replication studies with diverse ethnic groups and functional characterization are warranted to validate our findings.
